# Supplementary material for: Effectiveness of non-lethal predator deterrents to reduce livestock losses to leopard attacks within a multiple-use landscape of the Himalayan region
Source: PeerJ. 2020 Jul 24;8:e9544. doi: 10.7717/peerj.9544 (PMC7384438; doi:10.7717/peerj.9544)
Supplement: Supplemental Information 1 [file peerj-08-9544-s001.docx]

Supplementary Table S1 Summary of the model averaged estimates (generalized linear mixed models) with poisson structure for probability of livestock predation by leopard within a fine scale of 50 m radius around human settlements

| **Coefficients** | **Estimate** | **Standard error** | **Z value** | **Probability** |
| --- | --- | --- | --- | --- |
| Intercept | -0.930 | 1.022 | -0.409 | 0.399 |
| Presence of fox light | -1.067 | 0.371 | -2.876 | 0.004 |
| Proportion of scrub cover | 0.021 | 0.014 | 1.662 | 0.109 |
| Proportion of tree cover | 0.031 | 0.020 | 1.548 | 0.121 |
| Proportion of herb cover | 0.025 | 0.022 | 1.079 | 0.309 |
| Altitude | 0.001 | 0.001 | 0.136 | 0.891 |
