# Supplementary material for: Effectiveness of non-lethal predator deterrents to reduce livestock losses to leopard attacks within a multiple-use landscape of the Himalayan region
Source: PeerJ. 2020 Jul 24;8:e9544. doi: 10.7717/peerj.9544 (PMC7384438; doi:10.7717/peerj.9544)
Supplement: Supplemental Information 2 [file peerj-08-9544-s002.docx]

Supplementary Table S2 Summary of the model averaged estimates (generalized linear mixed models) with poisson structure for probability of livestock predation by leopard within a coarser scale of 500 m radius around human settlements

| **Coefficients** | **Estimate** | **Standard error** | **Z value** | **Probability** |
| --- | --- | --- | --- | --- |
| Intercept | 2.141 | 0.415 | 5.252 | 1.381 |
| Presence of fox light | -1.035 | 0.450 | -2.302 | 5.213 |
| Altitude | -3.8E-05 | 0.0003 | -0.115 | 1.273 |
| Nightlight | -0.011 | 0.039 | -0.278 | 1.004 |
| Area of non-forest | -5.9E-07 | 7.25E-07 | -0.808 | 0.751 |
| Area of Scrubland | 1.14E-06 | 4.05E-06 | 0.282 | 0.695 |
| Area of Open Forest | -8.3E-07 | 2.13E-06 | -0.399 | 1.045 |
| Area of Moderate Dense Forest | -3.3E-06 | 1.47E-06 | -2.237 | 3.602 |
| Area of Very Dense Forest | 3.7E-07 | 1.86E-06 | 0.187 | 0.405 |
