# Supplementary material for: Effectiveness of non-lethal predator deterrents to reduce livestock losses to leopard attacks within a multiple-use landscape of the Himalayan region
Source: PeerJ. 2020 Jul 24;8:e9544. doi: 10.7717/peerj.9544 (PMC7384438; doi:10.7717/peerj.9544)
Supplement: Supplemental Information 3 [file peerj-08-9544-s003.docx]

Supplementary Table S3 Summary of the model averaged estimates (generalized linear mixed models) with poisson structure for influence of livestock husbandry on probability of livestock predation by leopard within a fine scale of 50 m radius around human settlements

| **Coefficients** | **Estimate** | **Standard error** | **Z value** | **Probability** |
| --- | --- | --- | --- | --- |
| Intercept | 0.3914 | 0.459 | 1.138 | 0.398 |
| Presence of fox light | -0.962 | 0.415 | -2.323 | 0.021 |
| Number of household | 0.043 | 0.058 | 0.742 | 0.458 |
| Number of people | -0.013 | 0.034 | -0.384 | 0.701 |
| Enclosure | 0.214 | 0.437 | 0.479 | 0.647 |
| Number of livestock | -0.0005 | 0.006 | -0.126 | 0.858 |
| Number of domestic guard dog | 0.198 | 0.166 | 1.192 | 0.234 |
