# Supplementary material for: Effectiveness of non-lethal predator deterrents to reduce livestock losses to leopard attacks within a multiple-use landscape of the Himalayan region
Source: PeerJ. 2020 Jul 24;8:e9544. doi: 10.7717/peerj.9544 (PMC7384438; doi:10.7717/peerj.9544)
Supplement: Supplemental Information 4 [file peerj-08-9544-s004.docx]

**Data recorded within all experimental/control site**

1. GPS Location of site:
2. Name of village:
3. Altitude:
4. Number of regional guardians in the village:
5. Experimental/Control Site:
6. Leopard tracks/pugmarks/direct sighting within trails/village:
7. Number of households within 50 m radius of fox light/control site:
8. Number of domestic/guard dogs present within individual households:
9. Number of persons within individual households:
10. Number and type of livestock possessed per household:
11. Condition and type of livestock enclosure:
12. Percentage of herb, scrub, tree and barren land within 50 m radius of experimental/control site:
13. Number and type of livestock killed by common leopard:
14. Date and time of livestock kill:
